# NEDD8 Deamidation Inhibits Cullin RING Ligase Dynamics

**DOI:** 10.3389/fimmu.2021.695331

**Published:** 2021-08-17

**Authors:** Priyesh Mohanty, Kiran Sankar Chatterjee, Ranabir Das

**Affiliations:** National Center for Biological Sciences, Tata Institute of Fundamental Research (TIFR), Bangalore, India

**Keywords:** bacterial effector, deamidation, protein dynamics (molecular dynamics), NMR spectroscopy, Cullin RING E3 ligases, enteropathogenic *E. coli*, cycle inhibitory factor

## Abstract

Cullin-RING ligases (CRLs) are a significant subset of Ubiquitin E3 ligases that regulate multiple cellular substrates involved in innate immunity, cytoskeleton modeling, and cell cycle. The glutamine deamidase Cycle inhibitory factor (Cif) from enteric bacteria inactivates CRLs to modulate these processes in the host cell. The covalent attachment of a Ubiquitin-like protein NEDD8 catalytically activates CRLs by driving conformational changes in the Cullin C-terminal domain (CTD). NEDDylation results in a shift from a compact to an open CTD conformation through non-covalent interactions between NEDD8 and the WHB subdomain of CTD, eliminating the latter’s inhibitory interactions with the RING E3 ligase-Rbx1/2. It is unknown whether the non-covalent interactions are sufficient to stabilize Cullin CTD’s catalytic conformation. We studied the dynamics of Cullin-CTD in the presence and absence of NEDD8 using atomistic molecular dynamics (MD) simulations. We uncovered that NEDD8 engages in non-covalent interactions with 4HB/αβ subdomains in Cullin-CTD to promote open conformations. Cif deamidates glutamine 40 in NEDD8 to inhibit the conformational change in CRLs by an unknown mechanism. We investigated the effect of glutamine deamidation on NEDD8 and its interaction with the WHB subdomain post-NEDDylation using MD simulations and NMR spectroscopy. Our results suggest that deamidation creates a new intramolecular salt bridge in NEDD8 to destabilize the NEDD8/WHB complex and reduce CRL activity.

## Introduction

Ubiquitination is a post-translational modification that involves the sequential transfer of Ubiquitin (Ub) by E1, E2, and E3 enzymes onto the lysine residue of a substrate protein or another Ub molecule. E3 ligases catalyze the transfer of Ub from the E2~Ub thioester conjugate to the substrate. E3 may belong to RING or HECT classes. RING-E3s (~600) use a RING domain to catalyze the Ub transfer from the E2~Ub thioester onto the substrate lysine (1). A significant subset of RING E3 ligases (25-30%), known as Cullin-RING ligases (CRLs), tightly regulates the levels of various cellular substrates (2). CRLs are large, multi-modular machines comprising of an N-terminal domain (Cullin^NTD^) for substrate recognition and a C-terminal domain (Cullin^CTD^) to associate with the RING ligase Rbx1/2 (3). CRLs require the covalent attachment of a Ub-like protein - NEDD8, for catalytic activation (4, 5). The NEDDylation effect can be reversed by the COP9 signalosome, which cleaves NEDD8 to regulate CRL activity (6).

**Figure 1:**
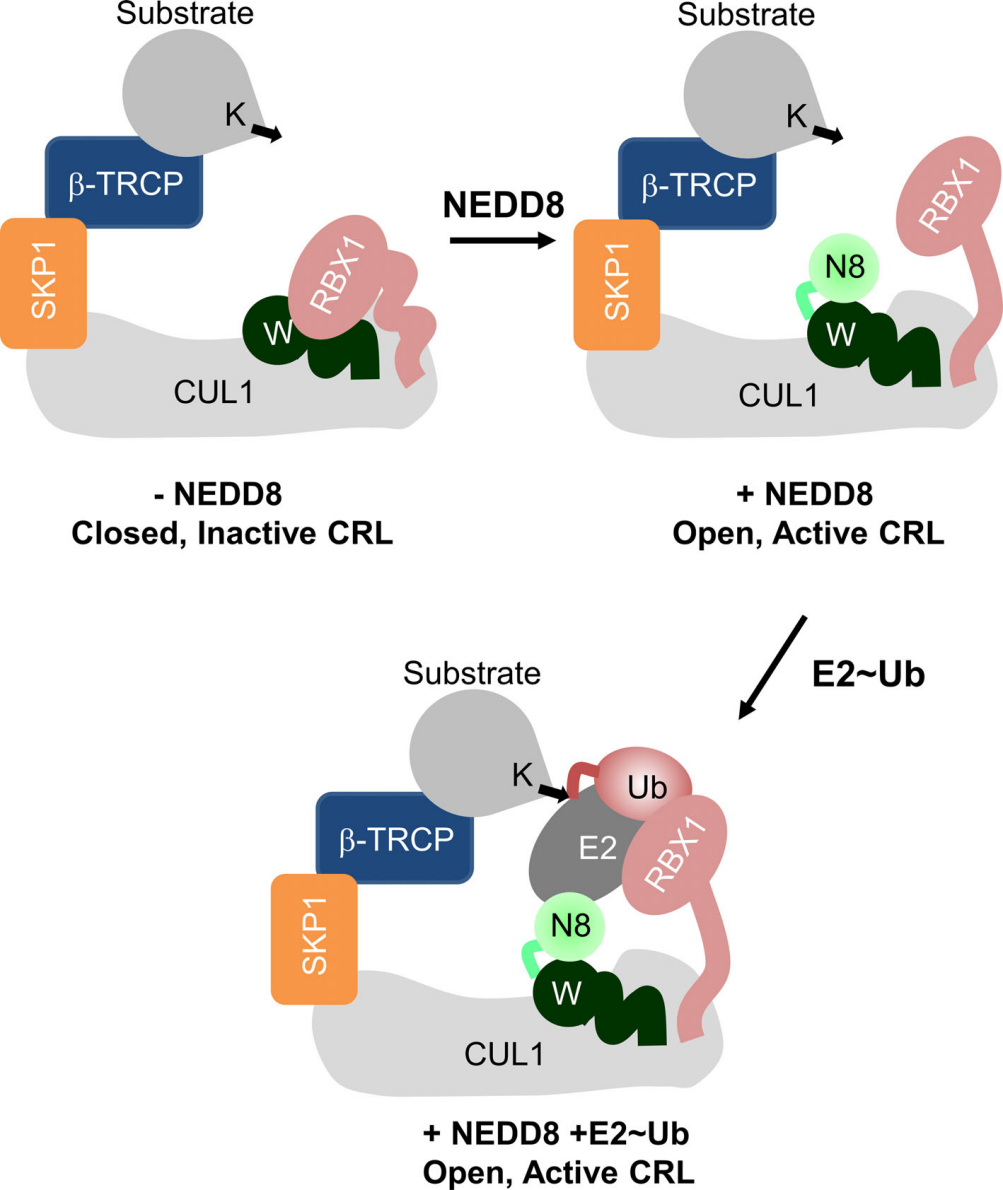
Role of NEDD8 in the activation of Cullin RING ligases. Schematic illustration showing the mechanism of Cullin RING ligase activation by NEDDylation. Conjugation of NEDD8 (N8) to the WHB^SD^ (W) triggers a change in its orientation which frees the Rbx^RING^ domain from inhibitory interactions.

Crystal structures suggest that NEDDylation promotes the Cullin^CTD^ transition from a closed, inactive state to an open, active state (7). Rbx1/2 interacts with the Winged-helix B subdomain (WHB^SD^) of Cullin^CTD^ in the closed, inactive form. Post-NEDDylation, NEDD8 engages in non-covalent interactions with the WHB^SD^ and masks its interaction with Rbx1/2. Moreover, it also triggers a domain rotation WHB^SD^ to promote interactions between WHB^SD^ and the four-helix bundle subdomain (4HB^SD^). Consequently, the Rbx RING domain (Rbx^RING^) transitions from a compact to an extended catalytic conformation (**Figure 1**). In silico models have proposed that in the full-length NEDDylated-Cullin, NEDD8 and Cullin^NTD^ may also interact (7). A recent structure of full-length, NEDDylated-CRL bound to E2~Ub conjugate reveals that NEDD8 interacts with Cullin^NTD^, Cullin^CTD^, and the E2~Ub to nucleate an active CRL/E2~Ub complex (8). However, the interaction dynamics between NEDD8 and Cullin in the absence of E2~Ub are unknown. These interactions are fundamental to the NEDD8-induced conformational change in CRL, which is the precursor to E2~Ub binding and activity.

Secreted bacterial effectors optimize the host cellular environment for replication. Glutamine deamidases convert glutamine to glutamate in their substrate proteins (9). The deamidase Cycle Inhibiting Factor (Cif) secreted by enteropathogenic *E. Coli* specifically deamidates the glutamine at position 40 (Q40) in NEDD8 to convert it to glutamate (10, 11). CRL facilitate the proteasomal degradation of CDK inhibitors p21/27, promoting timely progression through the G1/S and G2/M transition points of the eukaryotic cell cycle (2). Deamidated-NEDD8 (dNEDD8) drastically lowers the polyubiquitination activity of CRLs and blocks p21/p27 degradation, leading to cell cycle arrest (10). The Cullin family of E3 ligases also regulates the IκBα ubiquitination and degradation, activating the NFκB inflammatory responses (12). Cullin inhibition by NEDD8 deamidation could be instrumental in depleting the host inflammatory response. Cross-linking and Mass spectrometry experiments indicate that dNEDD8 prevents structural reconfiguration of Cullin^CTD^ necessary for CRL activation (13). A recent NEDD8~CUL1/Substrate/E2~Ub structure postulated that deamidation destabilizes the NEDD8/WHB^SD^ interface (8). A thorough understanding of how dNEDD8 prevents the structural reconfiguration in Cullin^CTD^ is currently lacking.

We report (i) NEDD8’s role in promoting Cullin-Rbx1 open/catalytic conformations and (ii) the mechanistic basis behind CRL inactivation by dNEDD8, using all-atom MD simulations and NMR spectroscopy. The NEDDylated-Cul5^CTD^ open conformation is stabilized by transient interactions between NEDD8 and 4HB/αβ-subdomains of Cullin^CTD^. NEDD8/Cullin^CTD^ interactions inhibit WHB^SD^’s tendency to associate with the Rbx1^RING^ domain and stabilize the closed conformation. NEDD8 deamidation results in an intramolecular salt-bridge formation, which competes with intermolecular interactions formed during NEDD8/WHB^SD^ association. Consequently, the dNEDD8/WHB^SD^ complex is unstable, and dNEDD8 cannot induce the CRL open/active conformation. Overall, this study provides valuable atomistic insights into NEDD8’s role in maintaining CRLs in an active conformation and the mechanism underlying its inhibition by bacterial deamidation.

## Methods

### Starting Structures and Molecular Modeling

Starting structures for MD simulations were obtained from the Protein Data Bank (PDB). Structures for closed and open conformations of Cul5^CTD^-Rbx1 were PDB id: 3DPL (chain: C/R) and PDB id: 3DQV (chain: A/B/C), respectively. The structure for dNEDD8 was taken from PDB id: 1NDD. Complexes of NEDD8~Cul1/5-WHB^SD^ were obtained from PDB id: 6TTU (Chain: C/N) and PDB id: 3DQV (Chain: A/C), respectively. NEDD8~Cul1-WHB^SD^ extended conformation was modeled from PDB id: 4P50 (chain A/K). Glutamine to glutamate substitutions in NEDD8 was introduced by replacing existing sidechains with best aligning rotamers from the Dunbrack rotamer library (14) in UCSF Chimera (15).

### MD Simulation Protocol

All systems except the extended conformation of NEDD8~Cul1-WHB^SD^ were parameterized using the AMBER99SB*-ILDN force field (16–18) with CUFIX (19–22) corrections. For extended NEDD8~Cul1-WHB^SD^, the AMBER99SBws (23) were used to prevent overestimation of protein-protein interactions and improve conformational sampling. All simulations were performed using the Gromacs 5.1.2 package (24, 25). Zinc coordination sites in the Rbx1^RING^ domain were modeled based on the Zinc AMBER force field (26) (ZAFF). The isopeptide bond between NEDD8-G76 and Cul1^CTD^-K721/Cul5^CTD^-K724 was modeled based on peptide bond parameters inAMBER99.

The starting structures were solvated in suitable cubic boxes by adding TIP3P/TIP4P2005 water molecules and 0.1 M NaCl. Cul5CTD-Rbx1 structures (closed/open) were simulated in a rhombic dodecahedral box with an edge length of 14.2 nm. dNEDD8 was simulated in a cubic box with an edge length of 6.5 nm. NEDD8/Cul1-WHB^SD^ association simulations were performed in a cubic box with an edge length of 12.0 nm. NEDD8~Cul1-WHB^SD^ complex variants were simulated in rectangular boxes with dimensions (nm): 9 x 8 x 8. Counter ions were added to neutralize the residual charge of the system. The electrically neutral system was then subjected to energy minimization using the steepest descent method for a maximum of 5000 steps until the maximum force on any atom was <1000 kJ mol^-1^nm^-1^.

Temperature and pressure equilibration was performed with harmonic positional restraints on all heavy protein atoms (k=1000 kJ mol^-1^nm^-1^) using periodic boundary conditions. Production MD simulations were carried out at 300 K and 1 bar pressure (NPT ensemble). Temperature control was achieved using the Berendsen thermostat (27) with a coupling constant (τt) of 2.0 ps. The Parrinello-Rahman barostat (28) was employed for pressure control using a coupling constant (τp) of 5 ps. All bond lengths were constrained using the LINCS (Hess, 2008) algorithm. Virtual interaction sites (29) were employed for hydrogen atoms, which permitted a 4 fs time step. The mass of water oxygen was reduced from 16 to 2 amu to improve sampling efficiency. Short-range electrostatics and van der Waals interactions were calculated using a 1.2 nm cutoff. Long-range electrostatics were calculated using Particle Mesh Ewald (30, 31) summation. SMD simulations of NEDD8~Cul5-WHB^SD^~NEDD8 complexes were carried out in a rectangular box of dimensions (nm): 12 x 9 x 9. Dissociation of the complexes was performed for 16 ns at a pull rate of 0.25 nm ns^-1^ using a moving harmonic potential (force constant = 1500 kJ mol^-1^ nm^-2^) applied to the NEDD8 (aa:1-70) COM. The COM motion of WHB^SD^ was removed every 100 fs to promote the build-up of the unbinding force.

### Structure and MD Trajectory Analysis

Nonbonded interactions in crystal structures were identified using the contact analysis tool in UCSF chimera. MD trajectories were analyzed using analysis tools available within the Gromacs package. Conventional MD trajectories were analyzed for snapshots saved at 200/240 ps intervals. ‘gmx gyrate’ was used to calculate the radius of gyration for various Cullin^CTD^-Rbx1 complexes. Inter-atomic distances and the number of contacts were analyzed using the ‘gmx mindist.’ ‘gmx hbond’ script was used to analyze hydrogen bonds. For SMD trajectories, force and COM separation values were recorded every 4 ps. Mean Force-time and work-time profiles were calculated over twelve independent trajectories to obtain mean F_max_ and W for enforced dissociation. Two-dimensional free energy landscapes for Cul5^CTD^-Rbx1 with and without NEDD8 were computed using a bin width of 0.03 nm, and the normalized free energy (ΔG) for each bin was determined using the relation:

$$\Delta G(R1,R2)=-k_{B}T[lnP_{i}-lnP_{max}],$$

where R1/R2 are reaction coordinates, k_B_ is the Boltzmann constant, T is the temperature, P_i_ is the joint probability of R1/R2 for a given bin, and P_max_ is the maximum probability. The lowest free energy state corresponds to ΔG = 0.

### Protein Expression and Purification

Plasmid for NEDD8 for bacterial expression was procured from Addgene. For N-terminal His-tag addition, the plasmid DNA was subcloned in Kanamycin resistance pet28b vector. Substitutions were done using Site-directed mutagenesis, and the corresponding clone was verified by sequencing. For overexpression and purification, clones were transformed in BL21 (DE3) bacterial cells and grown in an M9 medium containing ^15^NH_4_Cl and ^13^C-glucose. Cells were grown at 37°C, and protein expression was induced at OD_600_ of 0.8 by adding IPTG (isopropyl this-β-d-thiogalactoside) final concentration of 0.25mM. After five hours of further growth, the cells were harvested by centrifugation. NEDD8 and Q40E NEDD8 were purified from inclusion bodies by unfolding and refolding, according to the previously reported method (32). The cells were re-suspended in the lysis buffer [50 mM Tris, (pH 8.0), 300 mM NaCl], lysed by sonication. The lysate was centrifuged at 15000 rpm at 4°C, and the supernatant was discarded. The remaining inclusion bodies were washed and dissolved in denaturant buffer (8M Urea, 25 mM Tris, 150 mM NaCl) and sonicated further until the solution became clear. The solution was mixed with pre-equilibrated Ni^2+^ NTA-agarose beads (Protino) for 30 minutes. The slurry mixture (lysate with beads) was loaded to an open column, washed extensively with high salt lysis buffer for removing DNA impurities, and eluted with different imidazole concentrations present in denaturant buffer (pH 8.0). The eluted fraction was dialyzed overnight at 4°C in 0 M Urea buffer (25 mM Tris, pH 7.6, 150 mM NaCl) for refolding. Further purification was done by gel filtration (Superdex 75 16/600) column. The final protein was obtained in PBS containing 1 mM DTT at pH 7.4.

### NMR Spectroscopy

The NMR experiments were recorded at 298K on an 800 MHz Bruker Avance III HD spectrometer with a cryoprobe head. The samples were prepared in PBS with 1 mM DTT, pH 7.4. The protein sample was supplemented by 10% D2O. The standard triple resonance experiments HNCA and HN(CO)CA were used for assigning the chemicals shifts of ^13^C, ^15^N, and ^1^H backbone atoms. 95% (69 out of 73) of the non-proline backbone amide resonances were assigned in the protein. The assignment is deposited in BMRB with id 50948. Backbone assignment for wt NEDD8 was already available from BMRB (Entry 10062) (33).

## Results

### Both the apo-Cullin^CTD^ and NEDDylated-Cullin^CTD^ Are Dynamic

**Figure 2:**
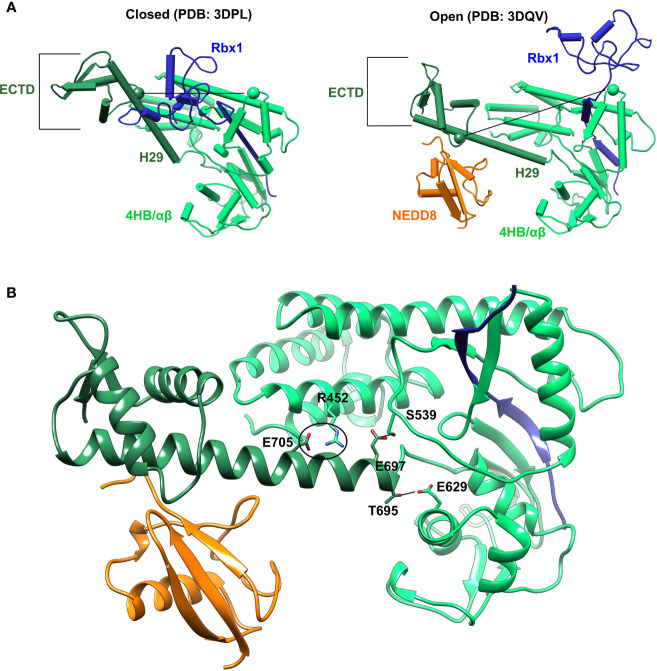
Crystal conformations of Cul5^CTD^ closed and open states. **(A)** Crystal structures of Cul5^CTD^-Rbx1 in the closed/open state were used to initiate independent MD simulations. Dotted black lines connect the Cα atoms (spheres) of S567 and R714 in Cul5^CTD^. ECTD in both complexes refers to the extreme C-terminal domain, which comprises aa:726-780. **(B)** Non-covalent interactions between WHB^SD^ and 4HB/αβ^SD^ in the open conformation of NEDD8~Cul5^CTD^-Rbx1 are shown in **(A)**. The black circle represents the R452/E705 salt bridge, while black lines correspond to S539/E697 and E629/T695 hydrogen bonds. The RING domain of Rbx1 (blue) is omitted for the sake of clarity.

Cullin C-terminal domain comprises of four-helix bundle (4HB), α/β, and winged-helix B (WHB) subdomains (**Figure 2A**). The open and closed Cul5^CTD^-Rbx1 structures suggest that NEDDylation drives a reorientation of the WHB^SD^ (**Figure 2A**). NEDD8 masks the Rbx1^RING^ interaction surface on WHB^SD^ (helix-29/ECTD) to promote the WHB^SD^ reorientation. Consequently, the WHB^SD^-Rbx1^RING^ interaction is disrupted, and Rbx1 adopts open and flexible/dynamic conformations, essential for the CRL activity (7). In the open conformation, helix-29 of WHB^SD^ interacts with 4HB/αβ^SD^. However, there are no short-range interactions between NEDD8 and 4HB/αβ^SD^ subdomains (**Figure 2B**). Moreover, unlike the closed conformation, there are no interactions between ECTD and 4HB^SD^ in the open conformation (**Figure 2B**). In the NEDD8~Cul5^CTD^ crystal structure, the open conformation appears to be stabilized through mutual interactions between two NEDD8~Cul5^CTD^-Rbx1 conformers in the asymmetric unit (**Figure S1**). Altogether, whether the NEDD8~Cul5^CTD^-Rbx1 open conformation observed in crystal structures represents a stable structure in solution is unclear.

**Figure 3:**
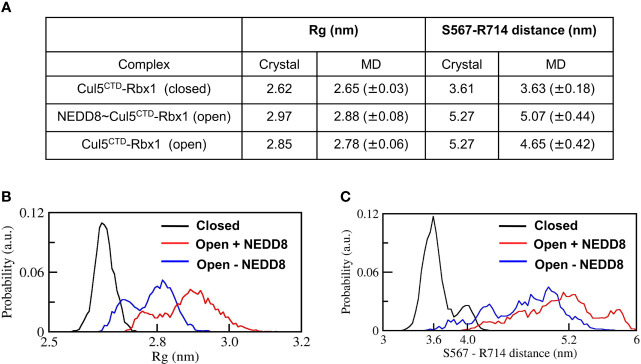
Dynamic ensembles of Cul5^CTD^ closed, and open states were observed from simulations. **(A)** Radius of gyration for Cul5^CTD^-Rbx1 variants and S567-R714 separation in Cul5^CTD^ calculated from MD simulations. Mean ± std deviation for each variant is calculated over eight independent MD trajectories performed for 200 ns. **(B)** Probability distributions for the radius of gyration (Rg) of closed/open states of Cul5^CTD^-Rbx1 calculated from the 1.6 μs macro trajectory for each state. The macro trajectory was obtained by combining eight independent 200 ns runs. **(C)** Probability distribution of S567-R714 distance in Cul5^CTD^ calculated from the 1.6 μs macro trajectory for each state.

The stability of Cul5^CTD^-Rbx1 open/closed conformations was studied using atomistic MD simulations with explicit solvent (Materials and Methods). The simulations were carried out both in the presence and absence of NEDD8. Overall dimensions of complexes were analyzed by their average radius of gyration (<Rg>), and their probability distributions were compared (**Figure 3**). <Rg> values calculated for the closed and open ensembles remain close to their crystal structures (**Figure 3A**). Simulations initiated from the closed conformation exhibited a narrow distribution of Rg about ~2.65 nm (**Figure 3B**). Simulations of the open conformation in the presence of NEDD8 showed a bimodal Rg distribution ranging from 2.7 to 3.1 nm. In the absence of NEDD8, the Rg distribution of the open conformation exhibited a shift towards closed-like conformations (**Figure 3B**).

The conformation of Cul5^CTD^ was analyzed by measuring the distance between Cα-atoms of two residues chosen for convenience, S567 and R714, which reduces from 5.3 nm to 3.6 nm as Cul5^CTD^-Rbx1 transitions from the open to closed conformation (**Figure 2A**). Simulations of the closed conformation generated a restricted Cul5^CTD^ ensemble, which was predominantly populated at an S567-R714 distance of ~3.6 nm (**Figure 3C**). In contrast, NEDD8~Cul5^CTD^-Rbx1 has an ensemble of Cul5^CTD^ conformations where the S567-R714 distance has an extended range of 4.0-6.0 nm (**Figure 3C**), which correlates well with its broad Rg distribution. NEDD8 removal causes a shift towards more compact Cul5^CTD^ conformations. The conformational heterogeneity observed in NEDD8~Cul5^CTD^ indicates that WHB^SD^ may adopt a range of orientations instead of a single orientation observed in the crystal structure. The multiple WHB^SD^ orientations result from frequent rupture of the short-range interactions that stabilize the orientation of WHB^SD^ against 4HB^SD^. The mean WHB^SD^/4HB^SD^ contact occupancies were merely 50%-75% during the simulation (**Figure S2**). Overall, the simulations underline the dynamic nature of the Cullin^CTD^ ensemble before and after NEDDylation. The dynamic Cullin^CTD^ ensembles may play a significant role in CRL activity by modulating its interaction with regulatory co-factors (34, 35).

### Transient Interactions Between NEDD8 and 4HB/αβ Subdomain Promotes the Extended Conformations of NEDD8~Cullin^CTD^

**Figure 4:**
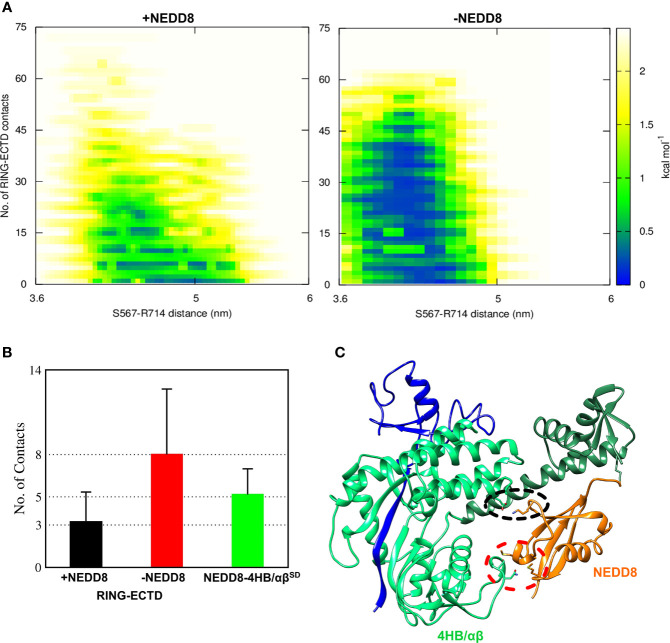
NEDDylation biases the conformational landscape of Cullin^CTD^-Rbx1 towards an ensemble of open conformations which minimize RING/ECTD interaction. **(A)** 2D-free energy landscapes calculated for the open state of Cul5^CTD^-Rbx1 with and without NEDD8 from 1.6 μs macro trajectories obtained by combining eight independent 200 ns trajectories for each state. Upon removal of NEDD8, ECTD/RING interaction is more favorable. **(B)** Mean + SEM for several ECTD/Rbx1^RING^ and 4HB/αβ^SD^-NEDD8 contacts across all Cul5^CTD^-Rbx1 trajectories initiated from the open state with and without NEDD8. Contact cutoff was chosen to be 0.4 nm. **(C)** A representative conformation of NEDD8~Cul5^CTD^-Rbx1 showing NEDD8 interacting with 4HB/αβ^SD^. Interacting atoms are shown as sticks. A black dotted circle indicates a salt bridge between NEDD8 (K48) and 4HB^SD^ (E407). The red dotted circle indicates van der Waals interactions between NEDD8 (M1/M62) and αβ^SD^ (N655/S656).

Although interactions between ECTD and Rbx1^RING^ are absent in the crystallographic open conformation, MD simulations indicate a tendency for such interactions to occur both in the presence and absence of NEDD8 (**Figures S3, S4**). Such interactions arise due to the dynamics of WHB^SD^ in the open conformation. NEDD8~Cul5^CTD^ was mostly open across all eight trajectories, and ECTD/RING interactions could be observed in only two trajectories (**Figure S3**). DeNEDDylation leads to compact Cul5^CTD^ for more extended periods, which increases the frequency of ECTD/RING interaction, as observed in four of the eight trajectories (**Figure S4**). The compact Cul5^CTD^ conformations stabilized by ECTD/RING interactions were observed in two of these trajectories (**Figure S5**). From the 2-D plots and the trajectories, it is clear that NEDD8 promotes extended conformations of Cul5^CTD^ to minimize ECTD/RING interaction (**Figure 4A** and **Movies S1**, **S2**). NEDD8~Cul5^CTD^-Rbx1 contact analysis indicates that NEDD8 had frequent contacts with 4HB/αβ^SD^ of Cul5^CTD^, including hydrogen bonds (**Figure S6**). The mean number of ECTD/RING contacts increased by more than two-fold upon removing NEDD8, indicating that NEDD8 inhibits the ECTD/RING interactions (**Figure 4B**). A representative NEDD8~Cul5^CTD^-Rbx1 conformation, wherein NEDD8 interacts with 4HB/αβ^SD^ is shown in **Figure 4C**. The occurrence of ECTD/RING interactions in NEDD8~Cul5^CTD^-Rbx1 simulations strongly suggests that partial masking of WHB^SD^ by NEDD8 is insufficient to maintain the open conformation. In summary, NEDDylated-Cullin^CTD^ exists as an ensemble of open conformations *via* transient interactions between NEDD8 and 4HB/αβ^SD^.

### NEDD8 Deamidation Creates a New Intramolecular Salt-Bridge

**Figure 5:**
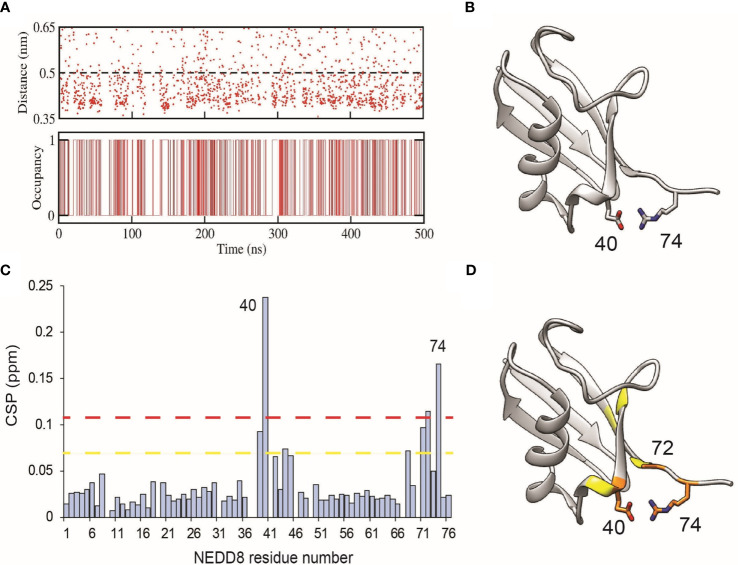
Formation of an intramolecular salt-bridge in dNEDD8. **(A)** Salt-bridge stability as a function of time in a 500 ns MD simulation of dNEDD8. In the bottom plot, salt-bridge occupancy is shown as either 1 or 0 to indicate the presence or absence of a salt-bridge, respectively. Salt-bridge occupancy was calculated using a 0.5 nm cutoff. The occupancy of the salt-bridge was calculated to be 44.2%. **(B)** MD snapshot showing the formation of R74-E40 salt-bridge in dNEDD8. **(C)** The NMR CSP plot showing the effect of Q40E substitution in NEDD8. The CSP = [(δH_NEDD8_ - δH_dNEDD8_)2 + (δN_NEDD8_ - δN_dNEDD8_)2/25]1/2, where δH_NEDD8_ and δH_dNEDD8_ are amide proton chemical shifts of residue in wt-NEDD8 and dNEDD8, respectively. The dashed yellow line and red line denote Mean+SD and Mean+2*SD, respectively. **(D)** The residues with high CSPs are mapped onto the NEDD8 structure. The residues with CSP above Mean+SD are colored yellow, and the residues with CSP above Mean+2*SD are colored orange.

NEDD8 deamidation disfavours open CRL conformations and reduces CRL activity. As a first step towards understanding how NEDD8 deamidation affects CRL activation, dNEDD8 (Q40E) was simulated for 500 ns. Intriguingly, an intramolecular salt bridge formed frequently between E40 and R74, located in the flexible C-terminal tail (**Figures 5A, B**). Solution NMR spectroscopy was used to probe the deamidation effect on NEDD8. Uniformly ^13^C, ^15^N labeled NEDD8, and dNEDD8 were grown and purified from E. coli. The dNEDD8 backbone amide resonances in the ^1^H-^15^N Heteronuclear Single Quantum Coherence (HSQC) NMR spectra were well separated, indicating that the molecule is folded (**Figure S7**). The chemical shifts of backbone amide resonances in NEDD8 were retrieved from previous data stored in the Biological Magnetic Resonance Bank (BMRB entry 10062) (33). The standard triple resonance experiments were used to assign the backbone amide chemical shifts in dNEDD8. An overlay of NEDD8 and dNEDD8 ^1^H -^15^N HSQC spectra shows minor changes in chemical shifts for a few backbone resonances (**Figure S7**). A chemical shift perturbation (CSP) plot revealed changes in two distinct regions in dNEDD8 (**Figure 5C**). The residues between 39 and 45 have significant CSP, with the highest CSP at E40 (**Figure 5C**), which is expected to be the deamidation site. Interestingly, the second set of residues affected by deamidation spans the C terminal tail in NEDD8. Residues 68 to 74 exhibit significant perturbation in their chemical shifts, with the highest CSP at 74 (**Figure 5C**). The high CSPs at these regions support the implication from MD studies that a new salt bridge is formed between E40-R74 dNEDD8 (**Figure 5D**).

### NEDD8 Deamidation Enhances Its Dissociation From WHB^SD^

**Figure 6:**
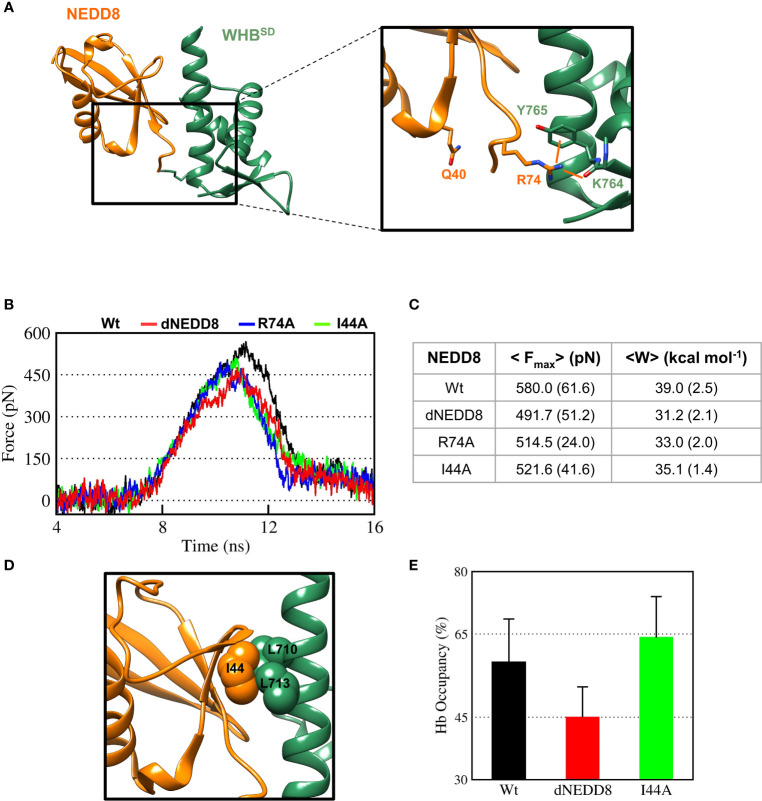
Deamidation of NEDD8 enhances its dissociation from WHB^SD^. **(A)** R74-mediated interactions in the Cul5-WHB^SD^~NEDD8 complex (PDB: 3DQV). Orange lines indicate interactions of R74 with and K764 (backbone) and Y765 (sidechain). **(B)** Mean force-time profile for the dissociation of NEDD8-wt and its mutants from WHB^SD^ obtained from steered MD (SMD) simulations. Twelve independent SMD runs were performed for 20 ns in the case of each complex. **(C)** F_max_ and unbinding work (W) determined from average profiles obtained by steered MD. One standard error of mean is indicated in ( ). **(D)** I44-mediated hydrophobic interactions in the Cul5-WHB^SD^~NEDD8 complex. **(E)** Mean occupancy of the R74-K764 hydrogen bond from NEDD8-wt, dNEDD8, and NEDD8-I44A SMD runs.

In the NEDDylated Cul5^CTD^-Rbx1 complex, R74 in NEDD8 interacts with K764/Y765, located in ECTD of WHB^SD^ (**Figure 6A**). The E40/R74 interaction in dNEDD8 could disrupt the intermolecular contacts of R74 and thereby destabilize the non-covalent complex. Steered MD simulations (12 independent runs) were performed to dissociate NEDD8 variants from WHB^SD^. The rupture force (F_max_) and cumulative work (W_unbind_) required for each variant’s dissociation were determined from these runs. As shown in **Figure 6B**, F_max_ and W_unbind_ were highest for the NEDD8 complex. Compared to NEDD8, F_max_ and W_unbind_ for dNEDD8 were reduced by ~90 pN and 8 kcal/mol, respectively, indicating a destabilized dNEDD8-WHB^SD^ complex (**Figure 6C**). I44 in NEDD8 engages in hydrophobic interactions with WHB^SD^ in the NEDD8-WHB^SD^ complex (**Figure 6D**). The F_max_ and W_unbind_ reduced when the I44 mediated contacts were disrupted (I44A substitution) (**Figure 6C**). However, dNEDD8 was more unstable compared to the I44A complex (**Figures 6B, C**). Moreover, destabilization of the dNEDD8 complex was comparable to the NEDD8-R74A complex, which suggested that E40 disrupted R74-mediated interactions during dNEDD8 dissociation.

Concomitantly, the R74/K764 hydrogen bond’s mean stability was reduced by 13-15% for dNEDD8 compared to NEDD8 or I44A-NEDD8 complex (**Figure 6E**). The R74/K764 hydrogen bond dynamics during the SMD in NEDD8 and dNEDD8 complexes are shown in **Figures S8, S9**. In a few dNEDD8 trajectories, transient salt-bridges (<0.5 nm) were observed between E40 and R74 from 8-16 ns (**Figure S9**), which appeared to compete with and destabilize the R74-K764 hydrogen bond. In conclusion, SMD simulations suggest that the intramolecular interaction between E40 and R74 in dNEDD8 may destabilize the NEDD8-WHB^SD^ complex.

### Intramolecular E40-R74 Interaction Interferes With the NEDD8/Cullin-WHB^SD^ Association

**Figure 7:**
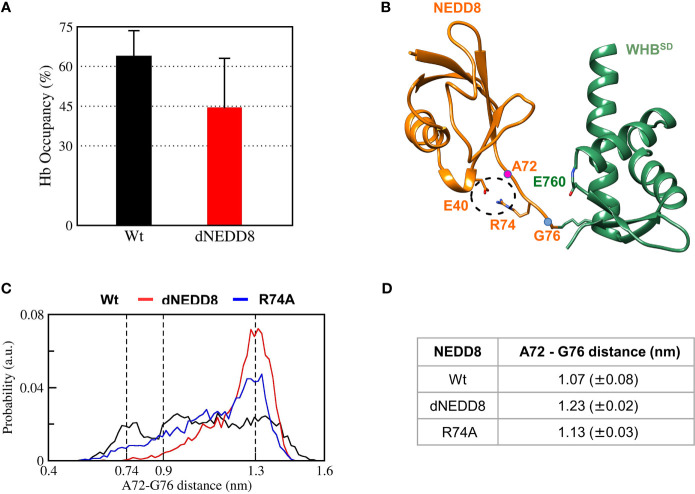
R74-E40 salt-bridge may hinder NEDD8/WHB^SD^ association by disallowing compact conformations of the NEDD8 C-terminal tail. **(A)** Mean± one standard error of R74/E760 hydrogen bond occupancy for NEDD8-wt and dNEDD8 conjugates. **(B)** Snapshot from trajectory 1 of Cul1-WHB^SD^~NEDD8 showing an intramolecular salt-bridge involving R74/E40. The Cα atom positions of A72/G76 are shown as pink spheres. **(C)** Combined probability distribution of the C-terminal tail conformation obtained from five 300 ns trajectories for each conjugate. The A72-G76 distance varies from 0.74 to 0.9 nm in crystal complexes of Cul1/5-WHB^SD^~NEDD8. **(D)** Mean ± one standard error of the C-terminal tail conformation across all five trajectories for each conjugate.

The E40-R74 interaction may also inhibit the association between dNEDD8 and WHB^SD^. An extended open conformation structure of NEDD8~Cullin-WHB^SD^ with no contacts between NEDD8 and WHB^SD^ is required as the starting structure to study the association, which is currently unavailable for Cul5 but available for Cul1. Hence, the NEDD8~Cul1-WHB^SD^ structure (PDB id: 4P5O) was chosen for this purpose (**Figure S10A**). Unbiased MD simulations were initiated from an extended NEDD8~Cul1-WHB^SD^ conformation to determine if E40 in dNEDD8 could compete for R74 during association with WHB^SD^. Five independent runs were performed (300 ns) for NEDD8 and dNEDD8-conjugated Cul1-WHB^SD^. For all NEDD8~WHB^SD^ variants, a range of extended conformations was observed across all trajectories with minimal interaction between NEDD8 (aa: 1-70) and WHB^SD^ (**Figure S10B**). In the NEDD8/dNEDD8 trajectories, R74 formed a hydrogen bond with E760, which corresponds to the same position as K764 in Cul5-WHB^SD^ (**Figure S11**). The mean occupancy of R74/E760 hydrogen bond calculated over NEDD8/dNEDD8 trajectories indicates a slight destabilization (>15%) for dNEDD8~WHB^SD^ due to competition with R74/E40 salt-bridge (**Figure 7A**). In dNEDD8 trajectories, E40 was found to compete for R74 in four of the five trajectories (**Figure S11C**). A representative WHB^SD^~dNEDD8 conformation with an E40/R74 salt-bridge is shown in **Figure 7B**. In the NEDD8~Cul1-WHB^SD^ and NEDD8~Cul5-WHB^SD^ complex structures, R74-mediated interactions stabilize the compact conformation of the C-terminal tail (**Figures 7A** and **S11A**), which likely reduces the range of motion for NEDD8 around WHB^SD^ and enhances non-covalent binding between NEDD8 and WHB^SD^. The conformation adopted by the C-terminal tail was determined by measuring the Cα distance between A72 and G76, which is 0.75 nm in the NEDD8~Cul5-WHB^SD^ crystal structure. The combined probability distributions of the C-terminal conformations from all wt-NEDD8, dNEDD8, and NEDD8-R74A trajectories are shown in **Figure 7C**. The probability distributions indicate that the C-terminal tail populates both compact and extended conformations to a similar extent in wt-NEDD8. In contrast, the dNEDD8 C-terminal tail has a strong bias towards extended conformations (**Figures 7C, D**). Similar to dNEDD8, NEDD8-R74A conjugate populates extended conformations of the C-terminal tail, confirming that R74-mediated hydrogen bonding is required for compact conformations. To summarize, R74-mediated hydrogen bonds with E760 of WHB^SD^ played a crucial role in NEDD8/WHB^SD^ non-covalent interaction. MD simulations suggest that intramolecular attraction between E40 and R74 in the dNEDD8~WHB^SD^ complex may disrupt R74 contacts with WHB^SD^ and destabilize dNEDD8~WHB^SD^ association.

### Deamidation Also Destabilizes the NEDD8~WHB^SD^/E2~Ub Complex

**Figure 8:**
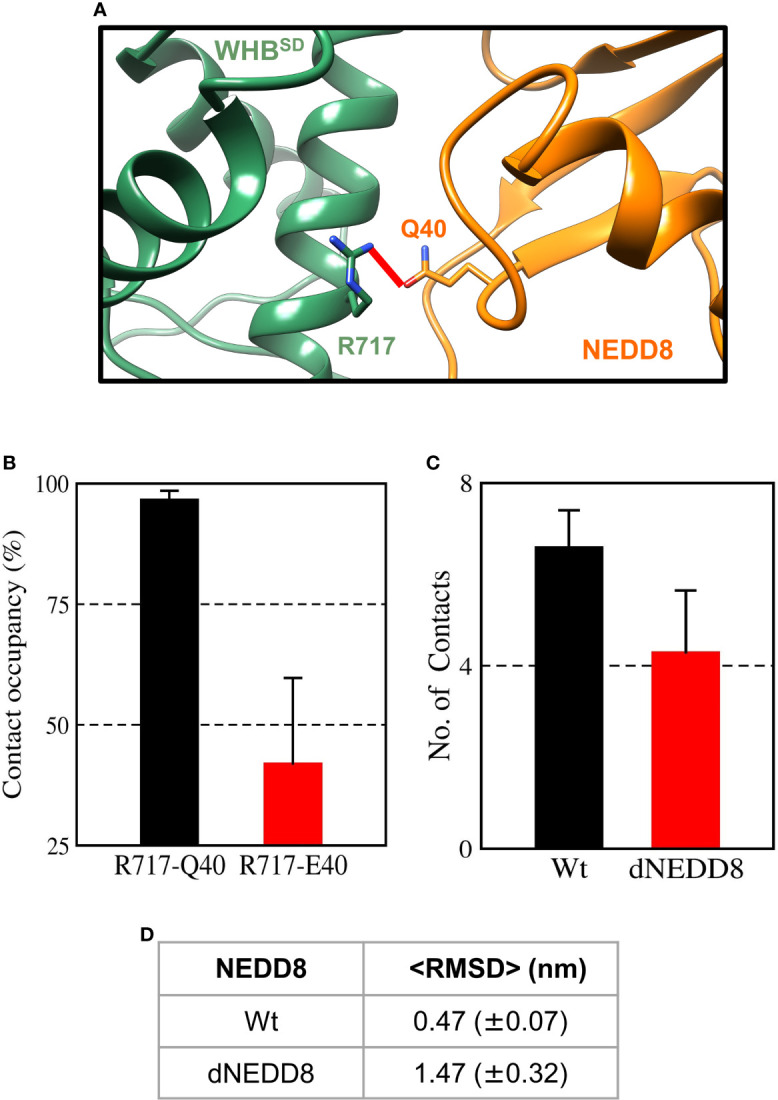
An intermolecular salt-bridge involving E40 destabilizes the Cul1-WHB^SD^~NEDD8 complex. **(A)** Relative positions of R717 and Q40 in the Cul1-WHB^SD^~NEDD8. The red line connects the Nη1 atom of R717 to the Oϵ atom of Q40 and has a length of 0.41 nm. The distance between the atoms drops below 0.32 nm in NEDD8-wt simulations, indicating a hydrogen bond formation. **(B)** Contact occupancies of R717-Q40 hydrogen bond, and R717-E40 salt-bridge were calculated using cutoffs of 0.32 and 0.5 nm, respectively. Mean ± std. Error is calculated for over three independent 200 ns trajectories. **(C)** Mean ± std error of the number of contacts formed between NEDD8 (I36/L71) and Cul1-WHB^SD^ (helix-29) sidechains using a cutoff of 0.45 nm. **(D)** Average RMSDs of Cul1-WHB^SD^~NEDD8 complexes over three independent 200 ns trajectories. ± One std. Error is indicated in ( ).

When E2~Ub binds to NEDD8~Cul1-Rbx1, NEDD8 is no longer associated with WHB^SD^ through its I44 patch (as in **Figure S11A**) (8). Instead, the I44 patch interacts with the ‘backside’ of E2. In this complex, NEDD8 Q40 is close to R717, located on helix-29 of WHB^SD^ (**Figure 8A**). The effect of deamidation was also investigated in the NEDD8~WHB^SD^ portion of this complex by simulations. The E2~Ub, Rbx1 & Cullin 4HB, α/β subdomains were removed before simulations to reduce the size of the system. In triplicate simulations, a stable hydrogen bond forms between Q40 and R717 across all trajectories (**Figures 8B** and **S12A**). Overall, the wt-complex maintained native hydrophobic interactions (**Figures 8C** and **S12C**) and had a mean RMSD below 0.5 nm (**Figures 8D** and **S12D**), indicating a stable complex. In contrast, the dNEDD8 complex was unstable, resulting in increased RMSD beyond 1 nm across all replicates (**Figure S12D**). Destabilization of dNEDD8 complex correlated with the lower contact occupancy of the R717-E40 contact (**Figures 8D** and **S12D**) and hydrophobic interactions (**Figure 8C**), which disrupted the orientation between NEDD8 and WHB^SD^, leading to an inactive Cul1-Rbx complex.

## Discussion

Our study provides atomistic insights into the role of NEDD8 in stabilizing the open, catalytically-active Cullin^CTD^ conformation and how its function is impaired by deamidation. Unlike a single conformation observed in crystal structures, NEDD8~Cul5^CTD^-Rbx1 exists as an ensemble of interconverting closed and open conformations. The orientation between WHB^SD^ and 4HB^SD^ observed in the crystal structure was unstable and adopted multiple orientations across independent simulations. The MD simulations suggest that NEDD8 activates Cullin^CTD^-Rbx1 through a combination of two mechanisms, (i) steric hindrance of interactions between Rbx1 and WHB/4HB^SD^, and (ii) transient interactions between NEDD8 and Cullin 4HB/αβ^SD^. The steric hindrance induces the open conformation. The transient interactions minimize ECTD/RING interaction, which also promotes the open/active conformation. These observations are supported by *in vitro* activity assays, which showed that in the absence of NEDD8, the ECTD deletion is sufficient to convert CRLs from an inactive to a constitutively active state (36).

Our results also uncover the underlying mechanism by which bacterial deamidation of NEDD8 inactivates CRLs. We have previously shown that the deamidation of the E2 enzyme-UBC13 by the *Shigella flexneri* deamidase – OspI triggers an intramolecular salt-bridge formation, inhibiting its association with the cognate RING E3 ligase - TRAF6 (37). We show here by MD simulations that deamidation at Q40 triggers the formation of an intramolecular salt bridge between E40 and R74. The NMR CSPs supported the observation. However, due to the low solubility of NEDD8 in *in-vitro* conditions, measurement of the salt bridge by hydrogen exchange or NOESY experiments was difficult.

The R74-E40 salt-bridge in dNEDD8 competes with an intermolecular hydrogen bond involving R74, which is required for stable association with WHB^SD^. Deamidation-induced competition for R74 promotes extended conformations of the C-terminal tail, inhibiting the formation of a stable NEDD8-WHB^SD^ complex. Even after E2~Ub interacts with CRLs, deamidation disrupts the Q40 contacts and destabilizes the NEDD8-WHB^SD^ complex. The inability of NEDD8 to associate with WHB^SD^ may effectively lock Cullin^CTD^-Rbx1 into a closed conformation, as suggested by XL/MS experiments (13).

Our results with Shigella flexneri deamidase OspI and E. coli deamidase Cif suggest a common inactivation mechanism of host cellular pathways by bacterial deamidases. The deamidated glutamine residue competes for functional electrostatic interactions between the target and its interacting partners. Disruption of these interactions adversely affects downstream signaling cascades and inhibits host immune responses to pathogen infection.

## Data Availability Statement

The original contributions presented in the study are included in the article/**Supplementary Material**. Further inquiries can be directed to the corresponding author.

## Author Contributions

PM carried out all MD simulations. KC performed the NMR experiments. RD supervised the project. PM and RD wrote the initial draft. All authors contributed to the article and approved the submitted version.

## Conflict of Interest

The authors declare that the research was conducted in the absence of any commercial or financial relationships that could be construed as a potential conflict of interest.

## Publisher’s Note

All claims expressed in this article are solely those of the authors and do not necessarily represent those of their affiliated organizations, or those of the publisher, the editors and the reviewers. Any product that may be evaluated in this article, or claim that may be made by its manufacturer, is not guaranteed or endorsed by the publisher.
