# Supplementary material for: NEDD8 Deamidation Inhibits Cullin RING Ligase Dynamics
Source: Front Immunol. 2021 Aug 17;12:695331. doi: 10.3389/fimmu.2021.695331 (PMC8418054; doi:10.3389/fimmu.2021.695331)
Supplement: Supplementary file 1 [file DataSheet_1.pdf]

NEDD8 deamidation inhibits Cullin RING ligase dynamics

Priyesh Mohanty[1], Kiran Sankar Chatterjee[1], and Ranabir Das[1*]

[1]National Center for Biological Sciences, TIFR, Bangalore, India

Supporting Information

*Electronic address: rana@ncbs.res.in

Legends for Supporting Movies

Movie S1. RING-ECTD interactions persist in Cul5^{CTD}-Rbx1 in the absence of NEDD8. The movie is based on a 200 ns trajectory of the Cul5^{CTD}-Rbx1 complex. The Cul5^{CTD}-Rbx1 complex is shown in ribbon representation. Rbx1 is colored in blue. 4-HB/αβ subdomains of Cul5 are colored in light green, while the WHB subdomain of Cullin is colored in dark green.

Movie S2. NEDD8 minimizes RING-ECTD interactions by engaging in non-covalent interactions with 4-HB/αβ subdomains of Cullin^{CTD}. The movie is based on a 200 ns trajectory of the NEDD8~Cul5^{CTD}-Rbx1 complex. NEDD8 is colored in orange. The color scheme for Cul5^{CTD} subdomains and Rbx1 is the same as in Movie S1.

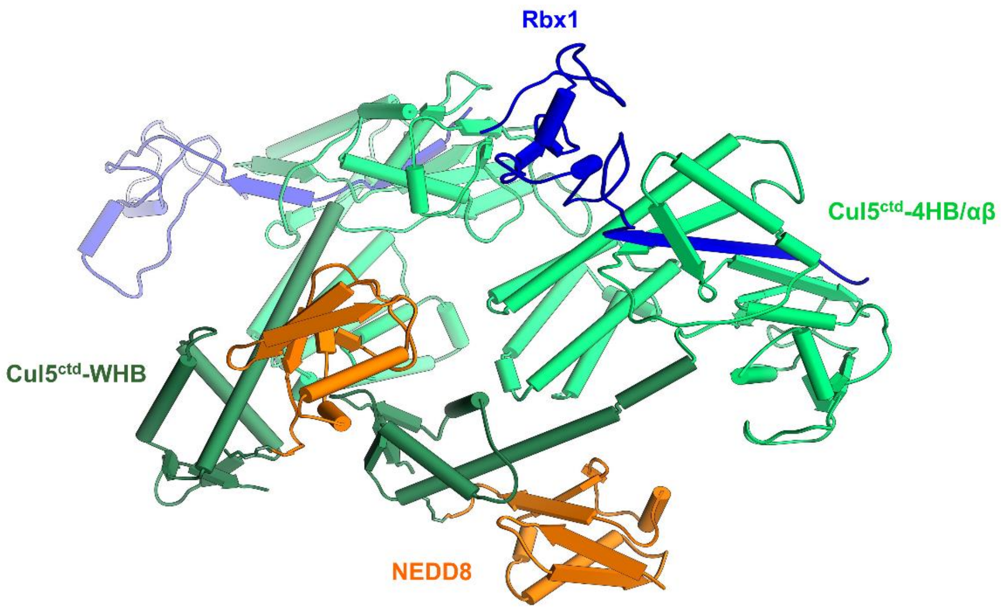

Figure S1. Stabilization of the open conformation of NEDD8~Cul5^{CTD}-Rbx1 in the asymmetric crystal unit. Related to Fig. 2. Two NEDD8~Cul5^{CTD} conformers in the asymmetric unit of PDB 3DQV showing an open state stabilized by mutual interactions.

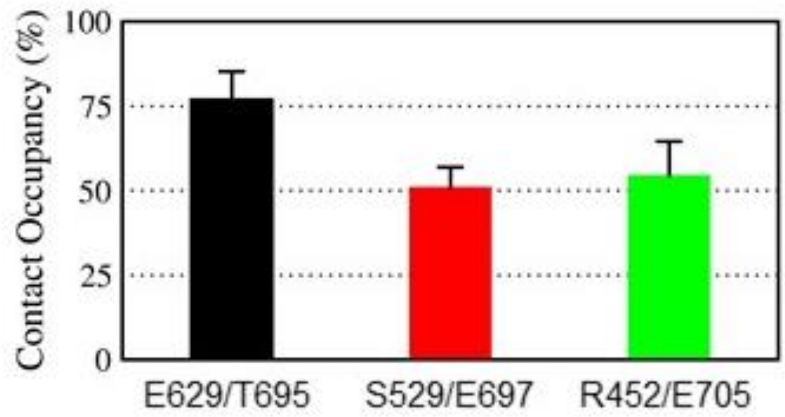

Figure S2. Mean occupancy of 4HB/αβ^{SD} -WHB^{SD} contacts across all NEDD8~Cul5^{CTD}-Rbx1 trajectories. Mean occupancies calculated for contacts are shown in Fig. 2B. For E629/T695 and S529/E697, distances were measured between Cδ and Oγ atoms. For R452/E705, the distance was measured between Cζ and Cδ atoms. Occupancies were calculated using a 0.5 nm cutoff.

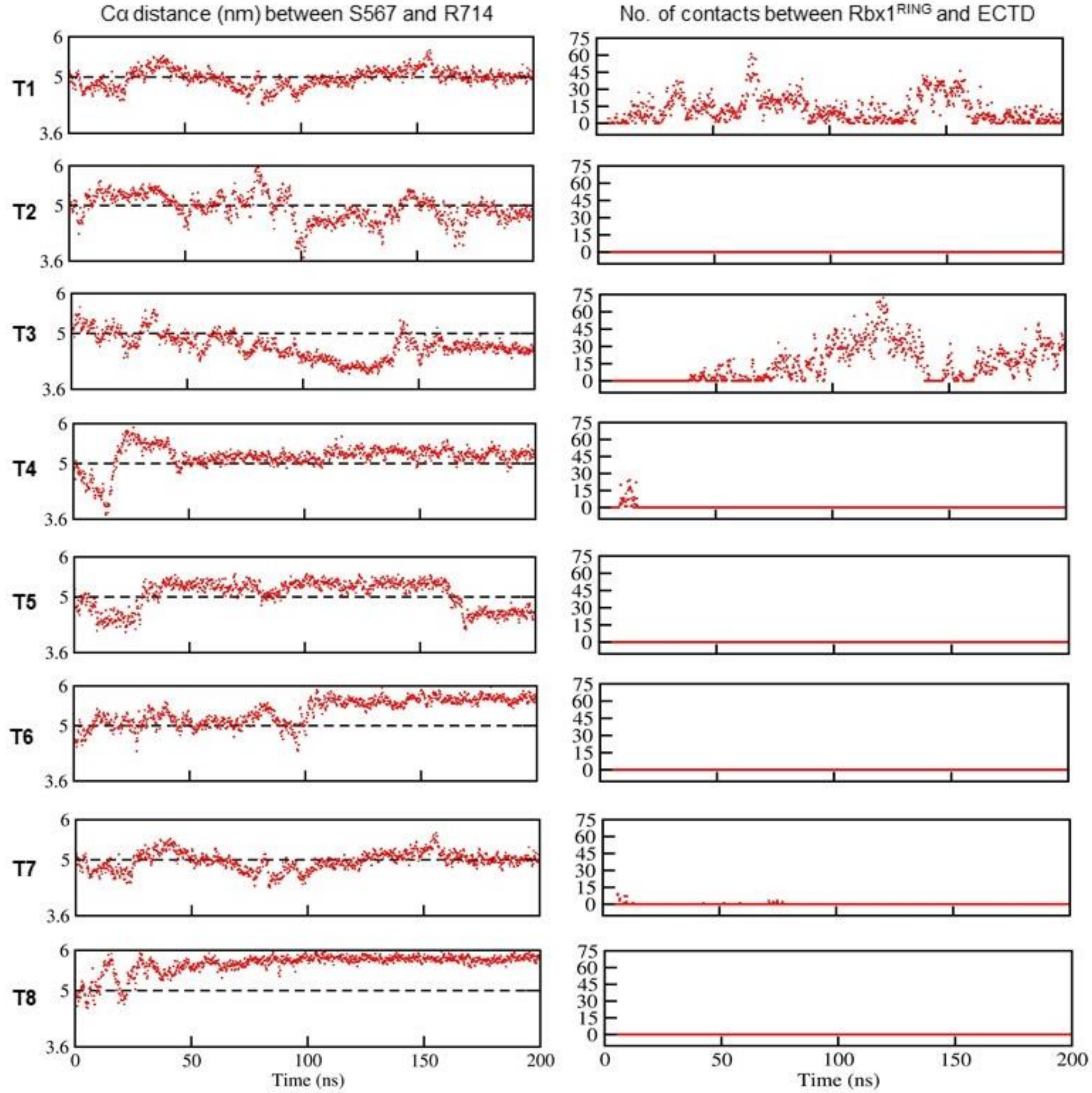

Figure S3. Conformational changes and RING-Cullin^{ECTD} interactions in NEDD8~Cul5^{CTD}-Rbx1 MD trajectories. Variation in S567-R714 Cα distance (left) and the corresponding variation in the Rbx1-RING/Cul5-ECTD contacts (right) across eight independent trajectories.

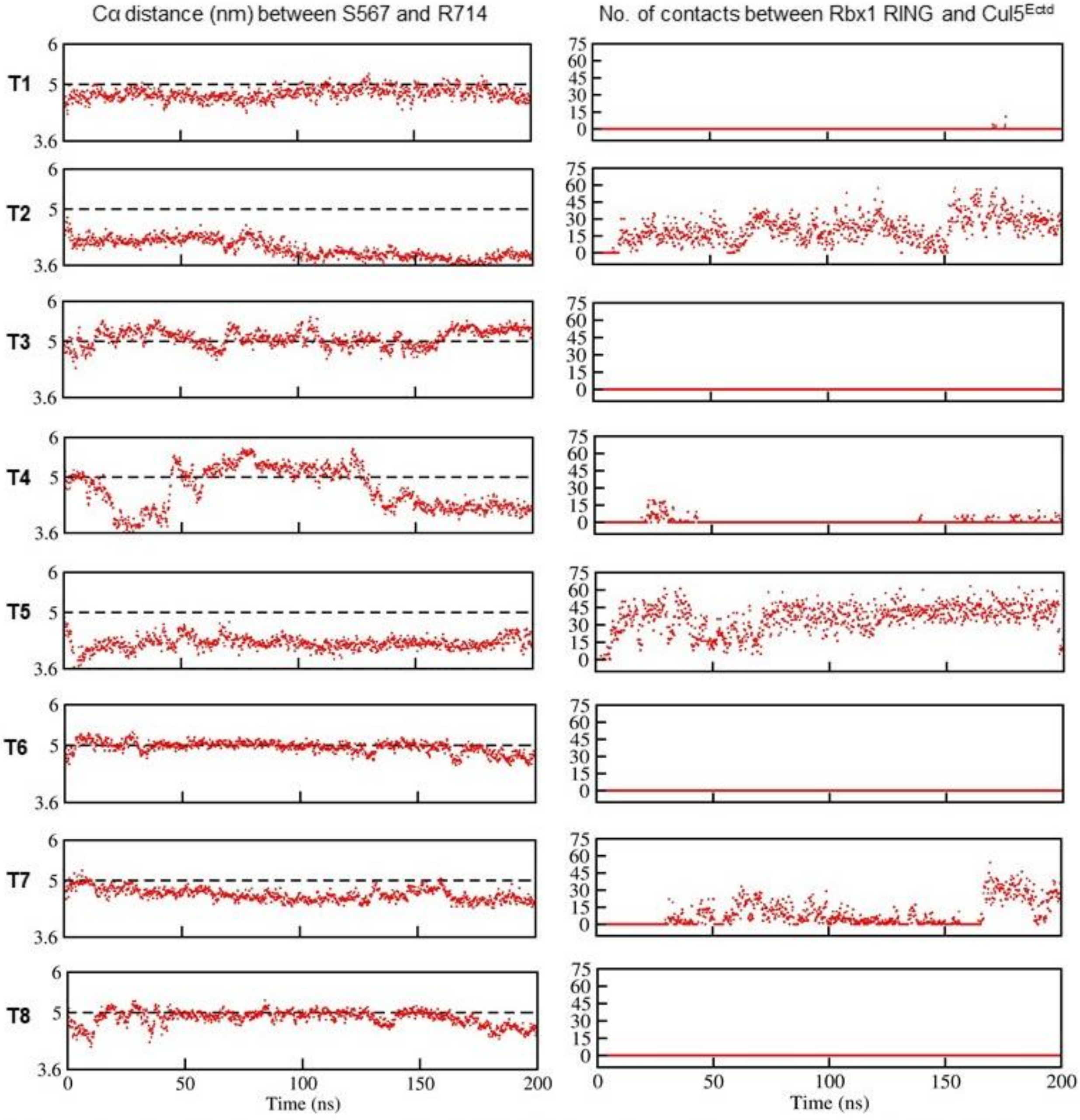

Figure S4. Conformational changes and RING-ECTD interactions in Cul5^{CTD}-Rbx1 (open) MD trajectories. Variation in S567-R714 Cα distance (left) and the corresponding variation in the number of Rbx1^{RING}/Cul5^{ECTD} contacts (right) across eight independent trajectories.

A

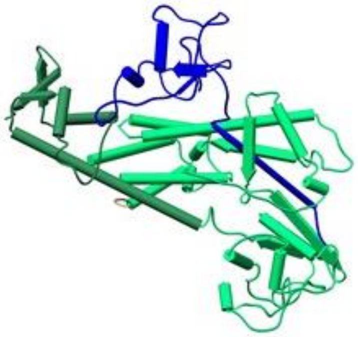

B

Rbx1-RING	Cul5-ECTD	No. of contacts
Ala63	Gln735	2
Ala63	Leu747	1
Glu66	Lys750	3

C

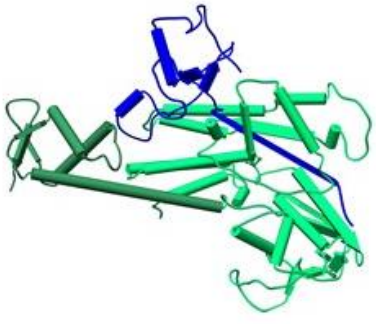

D

Rbx1-RING	Cul5-ECTD	No. of contacts
Leu52	Gln749	1
Leu52	Lys751	1
Cys56	Gln749	1
Cys56	Lys751	5
Gln57	Gln749	2
Gln57	Lys750	3
Gln57	Lys751	3
Ala58	Lys751	2
Ala58	Lys754	4
Gln60	Lys751	1
Gln60	Lys754	3
Gln60	Lys755	1

Figure S5. Conformational changes observed in MD runs initiated from open states of Cul5^{CTD}-Rbx1. A) and C) Representative conformations from two separate open state trajectories showing ECTD/RING interactions. B) and D) show the contacting residues and number of contacts in A) and C), respectively.

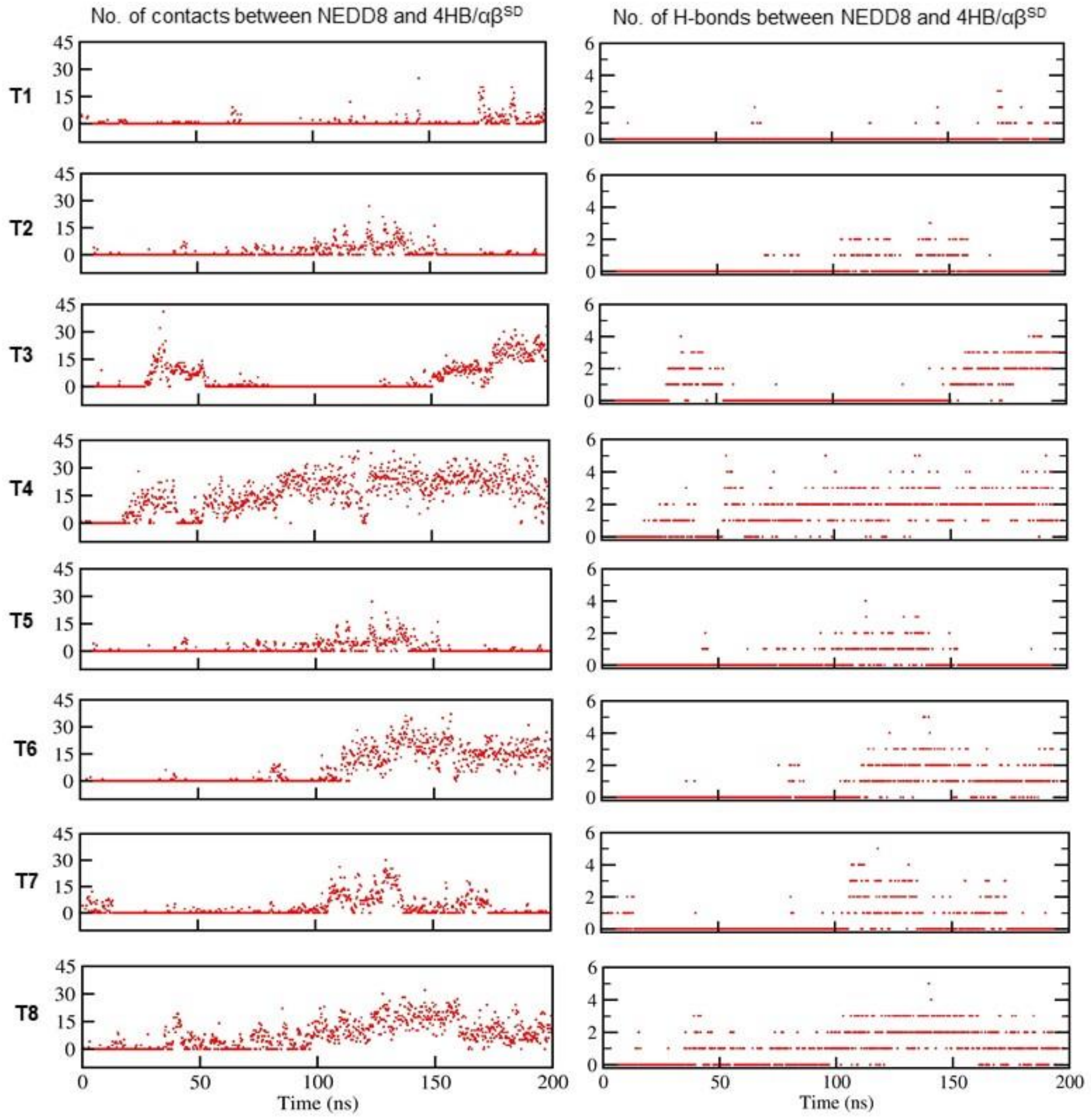

Figure S6. Analysis of interactions between NEDD8 and Cullin-4HB/αβ^{SD} observed in NEDD8~Cul5^{CTD}-Rbx1 trajectories. Variation in the total number of short-range contacts (left) and hydrogen bonds (right) between NEDD8 and 4HB/αβ^{SD} across eight independent trajectories. Contacts and hydrogen bonds were determined based on cutoffs of 0.4 and 0.35 nm, respectively.

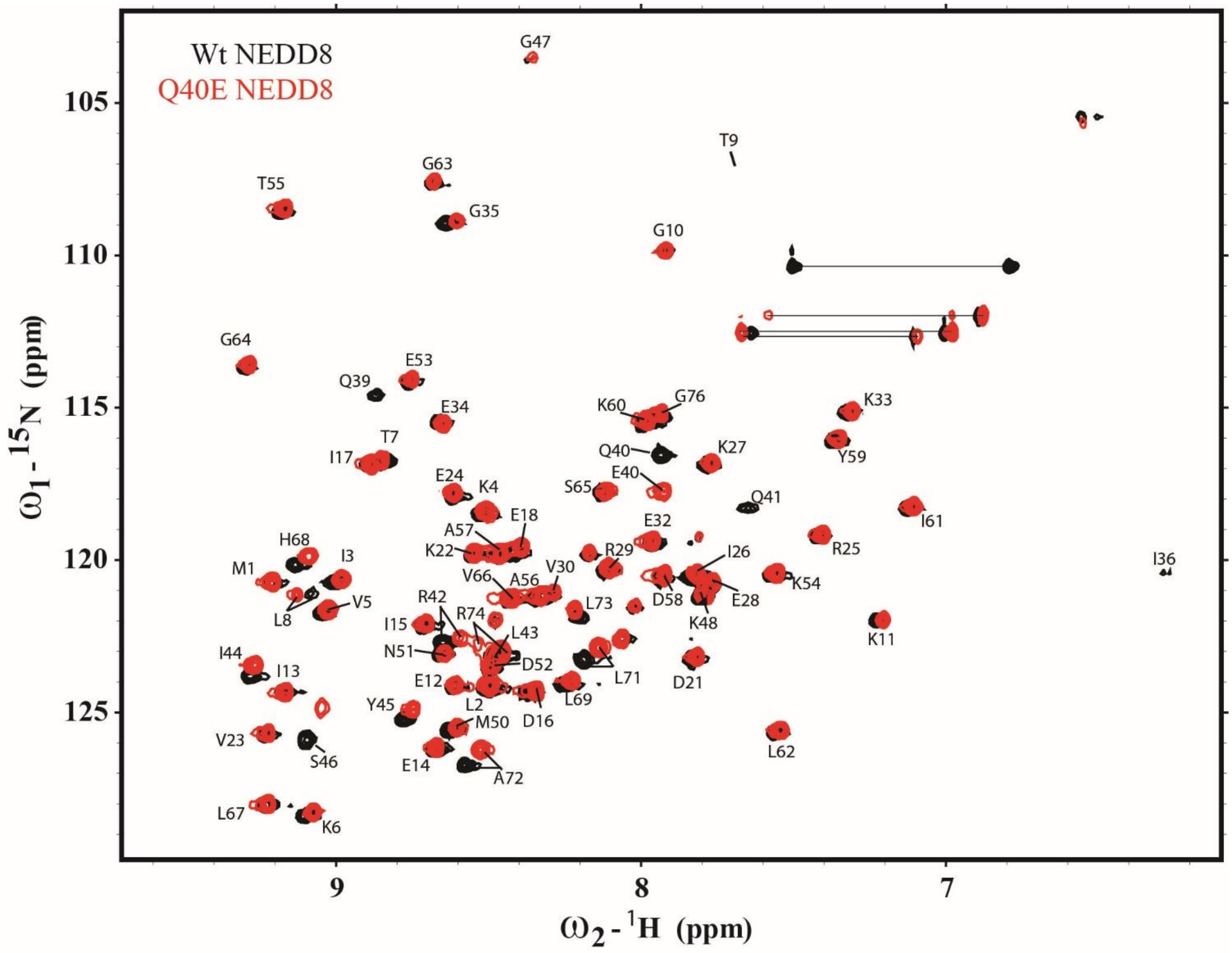

Fig S7. The NMR spectra of wt-NEDD8 and Q40E-NEDD8 (dNEDD8). The overlap ^1H-^{15}N-edited heteronuclear single quantum coherence spectra of wt-NEDD8 (black) and Q40E-NEDD8 (red). The black line connects sidechain amides of glutamines and asparagines. A few resonances like Q39, Q41, I36 in Q40E-NEDD8 are line broadened due to exchange, and their peak intensities were low. However, they could be assigned by the triple resonance experiments.

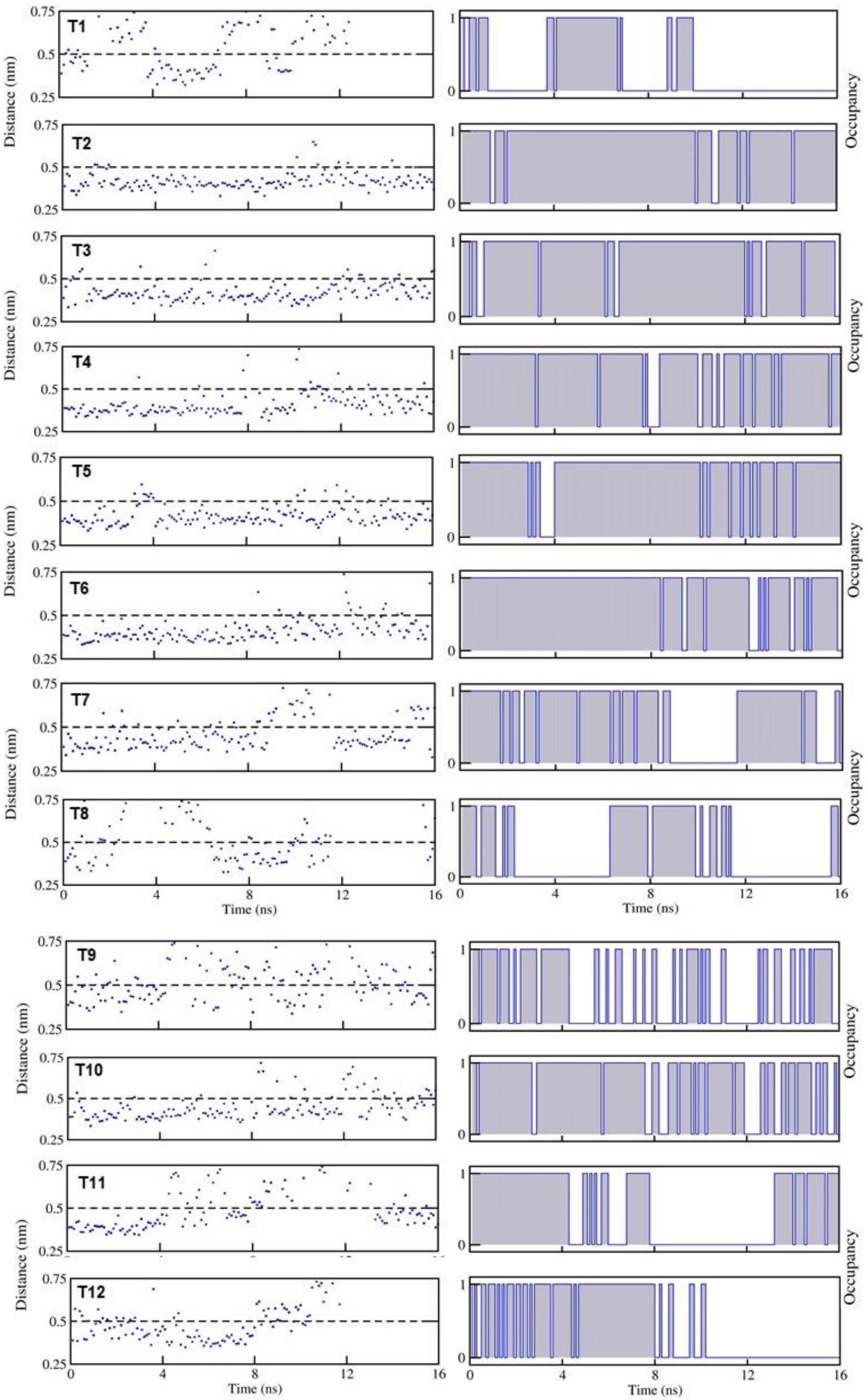
T1
T2
T3
T4
T5
T6
T7
T8
T9
T10
T11
T12
Distance (nm)
Occupancy
Time (ns)
0.25
0.5
0.75
0
4
8
12
16
1

Figure S8. Dynamics of R74/K764 hydrogen bonding from individual SMD runs for NEDD8. Distance variation as a function of time between R74-Cζ and K764-O atoms is shown on the left for 12 independent SMD trajectories (T1-T12). Distances below 0.5 nm correspond to potential hydrogen bonds. Right, hydrogen bond occupancy variation as a function of time (right) is derived from the distance plots where 1 corresponds to the presence of hydrogen bond and 0 corresponds to its absence.

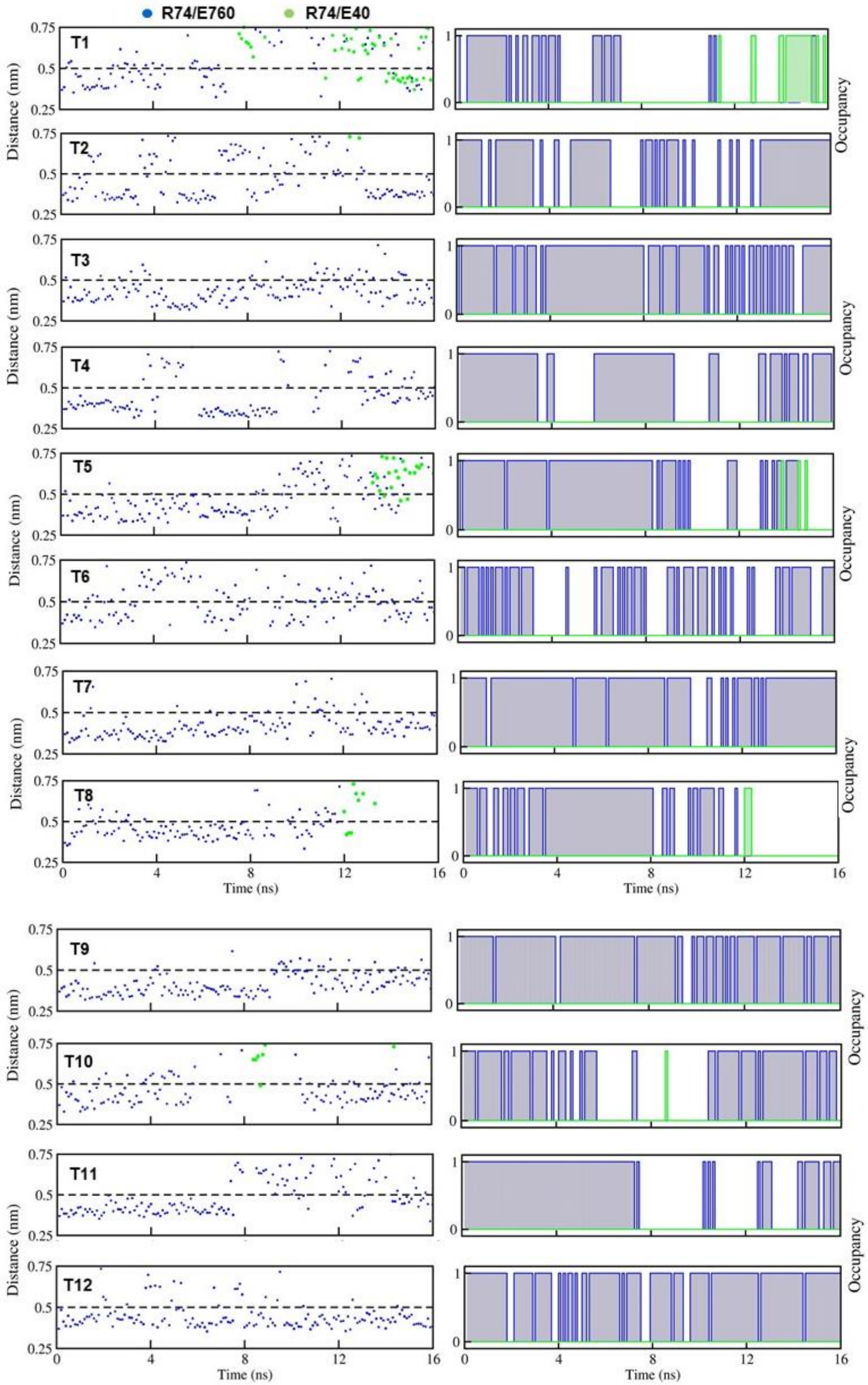

R74/E760
R74/E40
T1
T2
T3
T4
T5
T6
T7
T8
T9
T10
T11
T12
Distance (nm)
Occupancy
Time (ns)

Figure S9. Dynamics of R74/K764 hydrogen bond and R74/E40 salt-bridge formation from individual SMD runs for dNEDD8~WHB^{SD}. Distance variation as a function of time between R74-Cζ and K764-O/E40-Cδ atoms is shown for 12 independent SMD trajectories (T1 - T12) is provided in the left. Distances below 0.5 nm correspond to hydrogen bonds/salt-bridges. Right, hydrogen bond/salt-bridge occupancy variation as a function of time (right) derived from the distance plots where 1 corresponds to presence of a bond and 0 corresponds to its absence.

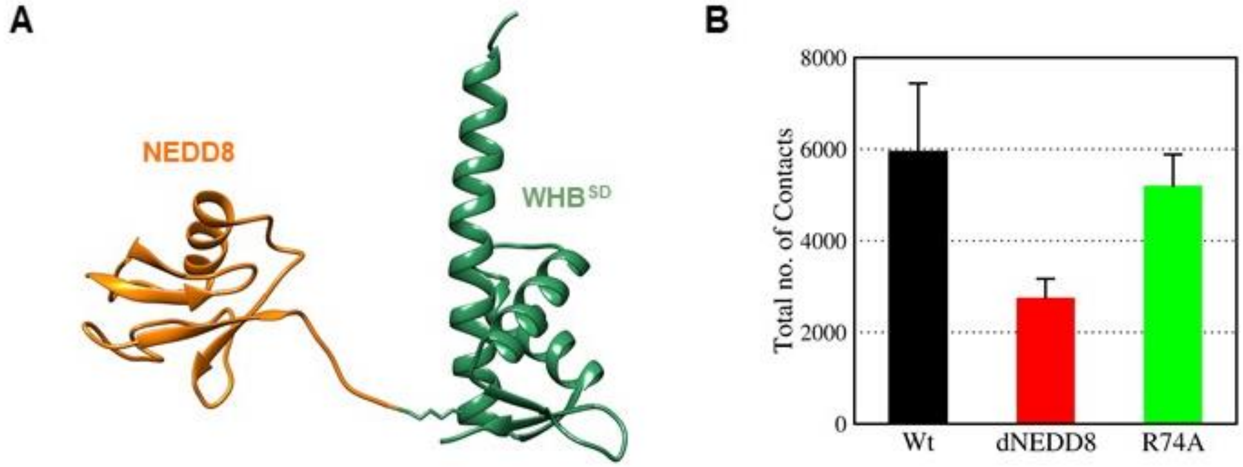

Figure S10. The extent of interaction between NEDD8 and WHB^{SD} in MD simulations initiated from the extended conformation. A) Extended conformation of Cul1-WHB^{SD}~NEDD8 modeled from PDB, which was used to initiate unbiased association simulations. B) Mean ± SEM for the total number of contacts formed between NEDD8 (aa: 1-70) and WHB^{SD} over five independent trajectories. Trajectories were analyzed at 240 ps intervals.

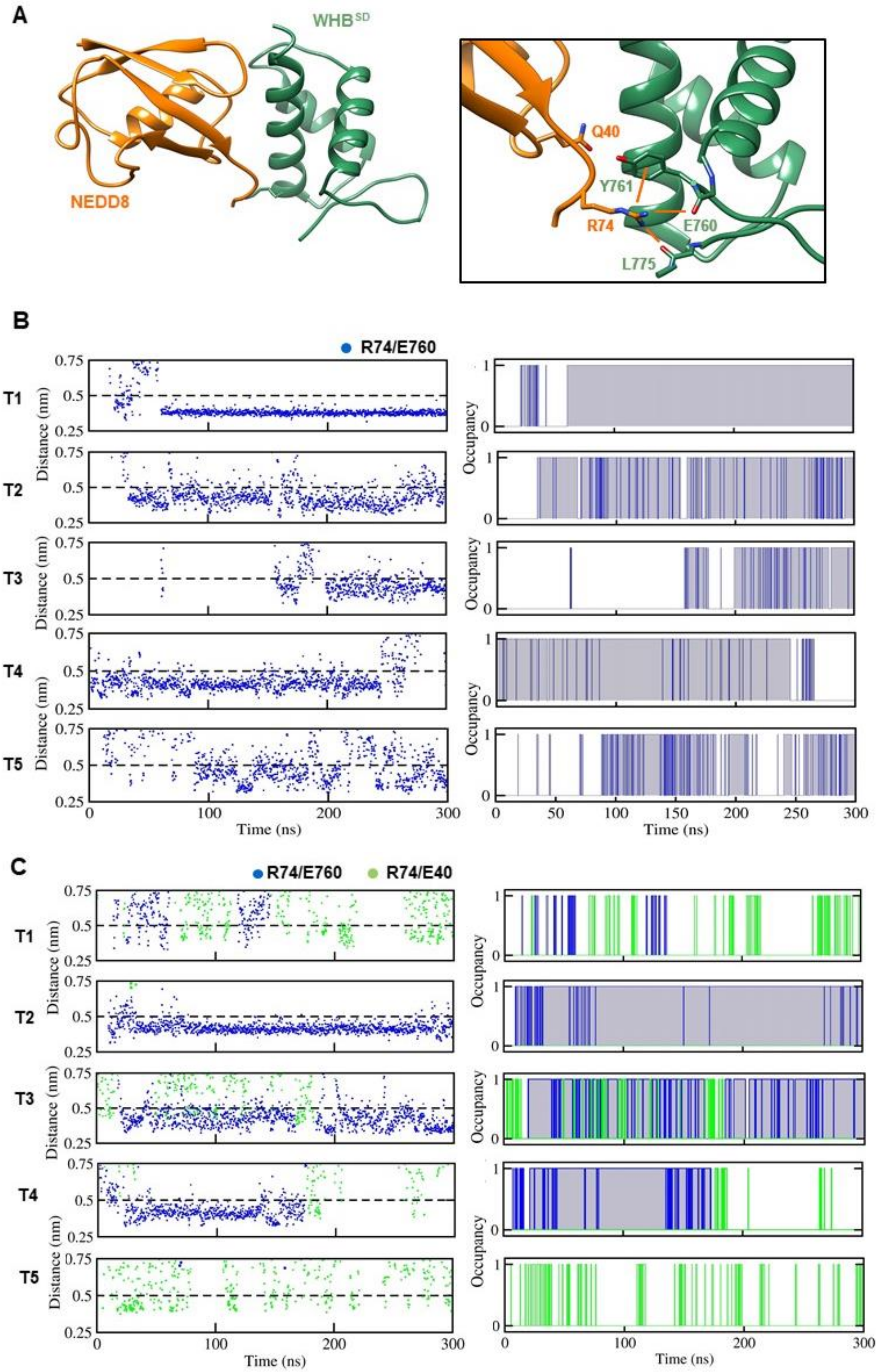

A
WHB^SD
NEDD8
Q40
Y761
R74
E760
L775
B
R74/E760
T1
T2
T3
T4
T5
Distance (nm)
Occupancy
Time (ns)
C
R74/E760
R74/E40
T1
T2
T3
T4
T5
Distance (nm)
Occupancy
Time (ns)

Figure S11. Dynamics of R74/E760 hydrogen bond and R74-E40 salt-bridge in extended NEDD8/dNEDD8 conjugates. A) R74-mediated interactions in the Cul1-WHB^{SD}~NEDD8 complex (PDB 6TTU). Orange lines indicate interactions of R74 with Y761 (sidechain) and E760/L775 (backbone). B) Variation in R74 and E760 atom distance as a function of time (left) and hydrogen bond occupancy (right) for NEDD8. Occupancy of 1 corresponds to a hydrogen bond, while 0 indicates its absence. Distances below 0.5 nm correspond to hydrogen bonds. C) Distance variation between R74-Cζ and E760-O/E40-Cδ (left) and hydrogen bond/salt-bridge occupancy for dNEDD8 conjugate. Distances below 0.5 nm correspond to hydrogen bonds/salt-bridges. In the dNEDD8 conjugate, E40 competes with E760 of WHB^{SD} by forming a salt bridge with R74.

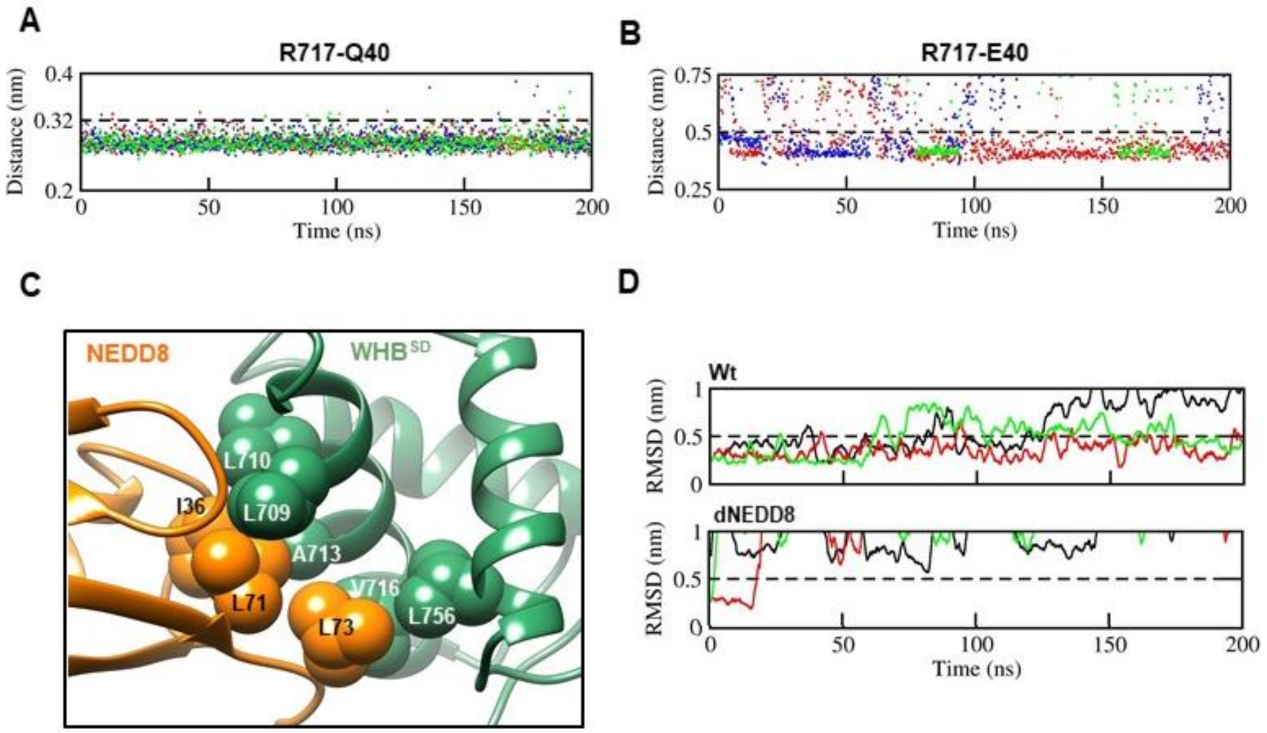

Figure S12. Non-covalent interactions stabilize the Cul1-WHB^{SD}~NEDD8 complex. A) The variation in distance between R717 Nη1 and Q40 Oε atoms in the NEDD8 complex in three replicate trajectories. Distances below 0.32 nm correspond to hydrogen bonds. Three replicate trajectories are colored red, blue, and green. B) The variation in distance between R717 Cζ and E40 Cδ atoms in the dNEDD8 complex in three replicate trajectories. C) Hydrophobic interactions in the Cul1-WHB^{SD}~NEDD8 complex. D) The variation in RMSD of NEDD8 and dNEDD8 complexes in triplicate trajectories, colored red, green, and black.
